# Response and Adherence to Nilotinib in Daily practice (RAND study): an in-depth observational study of chronic myeloid leukemia patients treated with nilotinib

**DOI:** 10.1007/s00228-020-02910-3

**Published:** 2020-06-02

**Authors:** Christel C. L. M. Boons, Lonneke Timmers, Jeroen J. W. M. Janssen, Peter E. Westerweel, Nicole M. A. Blijlevens, Willem M. Smit, Imke H. Bartelink, Janneke A. Wilschut, Eleonora L. Swart, N. Harry Hendrikse, Jacqueline G. Hugtenburg

**Affiliations:** 1 Department of Clinical Pharmacology and Pharmacy, Amsterdam UMC, Vrije Universiteit Amsterdam, Cancer Centre Amsterdam, De Boelelaan 1117, 1081 HV Amsterdam, The Netherlands; 2 Amsterdam Public Health Research Institute, Amsterdam UMC, Vrije Universiteit Amsterdam, De Boelelaan 1117, 1081 HV Amsterdam, The Netherlands; 3 Department of Hematology, Amsterdam UMC, Vrije Universiteit Amsterdam, Cancer Centre Amsterdam, De Boelelaan 1117, 1081 HV Amsterdam, The Netherlands; 4 Department of Hematology, Albert Schweitzer Ziekenhuis, Albert Schweitzerplaats 25, 3318 AT Dordrecht, The Netherlands; 5 Department of Hematology, Radboudumc, Geert Grooteplein Zuid 10, 6525 GA Nijmegen, The Netherlands; 6 Department of Hematology, Medisch Spectrum Twente, Koningsplein 1, 7512 KZ Enschede, The Netherlands; 7 Department of Radiology and Nuclear Medicine, Amsterdam UMC, Vrije Universiteit Amsterdam, Cancer Centre Amsterdam, De Boelelaan 1117, 1081 HV Amsterdam, The Netherlands

**Keywords:** Chronic myeloid leukemia, Nilotinib, Medication adherence, Patients’ experiences, Plasma concentration, Molecular response, Treatment outcome

## Abstract

**Introduction:**

This comprehensive observational study aimed to gain insight into adherence to nilotinib and the effect of (non)adherence on exposure (*C*_min_) and treatment outcomes.

**Methods:**

Chronic myeloid leukemia (CML) patients using nilotinib were followed for 12 months. Adherence was measured by Medication Event Monitoring System (MEMS), pill count, and Medication Adherence Report Scale (MARS-5). Nilotinib *C*_min_ and patient-reported outcomes (i.e., quality of life, side effects, beliefs, satisfaction) were measured at baseline, 3, 6, and 12 months.

**Results:**

Sixty-eight patients (57.5 ± 15.0 years, 49% female) participated. Median adherence to nilotinib (MEMS and pill count) was ≥ 99% and adherence < 90% was rare. Self-reported nonadherence (MARS-5) increased in the first year of treatment to a third of patients. In line with the strong beliefs in the necessity of taking nilotinib, forgetting to take a dose was more prevalent than intentionally adjusting/skipping doses. Nilotinib *C*_min_ were generally above the therapeutic target in 95% of patients. Patients reported a variety of side effects, of which fatigue was most frequent. The mean *C*_min_ was higher in patients who reported severe itching and fatigue. The overall 1-year MMR rate ranged from 47 to 71%.

**Conclusion:**

Substantial nonadherence (< 90%) to nilotinib was rare and nilotinib *C*_min_ were generally above the therapeutic target. Lack of response in our group of patients was not related to nonadherence or inadequate *C*_min_. Nevertheless, a considerable number of patients experienced difficulties in adhering to the twice daily fasted dosing regimen, emphasizing the importance of continuous support of medication adherence in CML.

**Clinical trial registration:**

NTR3992 (Netherlands Trial Register, www.trialregister.nl)

**Electronic supplementary material:**

The online version of this article (10.1007/s00228-020-02910-3) contains supplementary material, which is available to authorized users.

## Introduction

Newly diagnosed patients with chronic-phase chronic myeloid leukemia (CP-CML) are treated with tyrosine kinase inhibitors (TKI) including imatinib, dasatinib, nilotinib, or bosutinib which specifically block BCR-ABL activity [1]. Dasatinib, nilotinib, and bosutinib are so-called second-generation TKIs (2G-TKIs) that are more potent than imatinib. Clinical study data show that the overall survival of CP-CML patients achieving a major molecular response (MMR) on TKI treatment now resembles that of the general population [2, 3]. However, side effects like fatigue, edema, skin toxicity, accelerated cardiovascular disease, pleural effusion, and pulmonary hypertension may cause significant morbidity and reduce quality of life [4–6].

CP-CML treatment discontinuation may be attempted in patients attaining a deep molecular response at the level of MR^4^ or better [1, 7]. However, not all patients achieve these response levels. Causes include the presence of point mutations in the kinase domain of the BCR-ABL protein, increased BCR-ABL protein expression, and increased drug efflux mechanisms [8, 9]. In addition, pharmacokinetic factors like interindividual variabilities in the liver (CYP3A4) metabolism and plasma protein binding, or interactions with other drugs, may lead to suboptimal plasma drug concentrations that affect response rates [10–16]. In this respect, lower trough plasma concentrations (*C*_min_) have been associated with a failure to achieve key response milestones and a shorter time to progression in both newly diagnosed and imatinib-resistant or imatinib-intolerant CP-CML patients [12–15]. On the other hand, supratherapeutic TKI levels have been shown to increase toxicity. For example, high nilotinib peak plasma concentrations that may occur when the drug is taken with food may increase the QTc interval [16].

Next to these disease-related and pharmacokinetic factors, a major factor to treatment failure is medication nonadherence [17–22]. Several studies show that for imatinib, an adherence rate (mostly expressed by the Medication Possession Ratio [MPR]) of at least 90% is required to achieve an adequate (molecular) response and that nonadherent patients have a significantly reduced chance of reaching key response milestones [17–22]. For the 2G-TKIs, data on the minimum adherence required to achieve the intended medication effect are not available. The majority of data on adherence to 2G-TKIs have been obtained in retrospective studies [23–35], with only one study investigating adherence prospectively [36]. The average adherence rates range from 69 to 100% [23–36]. A wide variety of (interrelated) factors is known to influence adherence in CML. They include the choice of the medication, convenience of the dosing regimen, treatment duration, treatment effectiveness, occurrence of side effects affecting quality of life, patient beliefs, patient knowledge and understanding, disease characteristics (e.g., disease severity, comorbidity and comorbidity treatment, mental status), life style, communication with healthcare providers (HCP), and affordability of treatment [37–39]. Clearly, a better understanding of the variables affecting (non)adherence is necessary to optimize treatment conditions to a level that enables patients to use their prescribed medication as long as required while preserving the highest possible quality of life [37, 38, 40].

Most CP-CML patients have to take their oral medication indefinitely. In this respect, for patients taking nilotinib, the necessary twice daily fasted schedule is an additional burden that may trigger nonadherence. The RAND study (Response and Adherence to Nilotinib in Daily practice) was conducted with the aim to gain insight into nilotinib treatment adherence in daily clinical practice and the effect of (non)adherence on nilotinib exposure and treatment outcomes. Multiple measures were used to assess adherence including electronic monitoring, pill count and questionnaires, and factors influencing adherence including patient characteristics, side effects, quality of life, attitudes towards disease and treatment, and satisfaction with information were studied. Furthermore, we evaluated nilotinib exposure and its relationship with side effects and treatment outcome.

## Methods

### Study design

In this multicenter prospective observational study [41], conducted between August 2013 and April 2017 in six Dutch hospitals, CP-CML patients using nilotinib were followed for 12 months. Initially, it was the aim only to include newly diagnosed CP-CML patients starting nilotinib treatment. However, because of poor participant accrual, the protocol was amended in September 2014 and patients on treatment with nilotinib (and regardless of prior TKI treatment) were also included. The study was registered in the Netherlands Trial Register (NTR3992) and the protocol has been published previously [41].

### Patients

The study population consisted of patients (aged ≥ 18 years) with CP-CML using nilotinib [42]. According to the line of treatment (first- or second/third-line) and the type of nilotinib treatment at baseline (starting treatment or already being on treatment), four groups of patients were distinguished. Exclusion criterion was the inability to grant consent.

### Data collection

#### Medication adherence

Medication adherence has been defined as “the process by which patients take their medications as prescribed”, divided into three phases: initiation, implementation, and discontinuation [43]. The RAND study focused on the implementation phase and used three methods to assess adherence to nilotinib.

First, adherence was assessed by means of a medication event monitoring system (MEMS; AARDEX Group, Switzerland). Capsules were stored in a bottle closed by a lid containing the MEMS monitoring the date and time of opening. Each opening was presumed to indicate the intake of a dose, with a maximum of two intakes per day. Patients knew that dosing data were compiled by means of the MEMS. To minimize bias, patients were instructed that nilotinib use in general was studied and the study aim of measuring adherence was deliberately omitted in the patient information. Adherence was expressed as the proportion of days covered (PDC) and calculated by the number of times that the bottle had been opened divided by the total number of intakes prescribed over the period that the MEMS was used times 100%.

Second, adherence was assessed by means of pill count. Patients were contacted unannounced by the researcher by phone at study entry and at the end of the follow-up period to count the number of capsules at that moment. Patients were asked whether they had returned capsules to the pharmacy or disposed capsules in any other way. Pharmacy dispensing records and the prescribed number of capsules retrieved from the patient’s medical file were collected. Adherence was expressed as the adherence rate (AR) and calculated by dividing the number of capsules dispensed minus the pill count by the number of capsules prescribed over the study period times 100% [33].

Third, the five-item Medication Adherence Report Scale (MARS-5) was used to assess self-reported adherence [44–46]. The MARS-5 was administered at 3, 6, and 12 months from study entry. Items are scored using a 5-point scale (1 = always to 5 = never). Scores for each item were summed to give a total score ranging from 5 to 25, with higher scores indicating higher levels of adherence. A self-composed question assessed nilotinib intake in relation to food intake. Nilotinib intake under fasting conditions was scored as incorrect when patients reported to have taken nilotinib < 60 min before or < 120 min after food intake.

#### Trough plasma concentration

Blood sampling was performed by the patient at home by means of a validated dried blood spot (DBS) sampling method. Using the DBS, nilotinib plasma concentrations were calculated as previously reported [47, 48]. Patients were instructed to sample blood at predose at baseline (if already on nilotinib treatment), 3, 6, and 12 months from study entry. The time of blood sampling, time of last nilotinib intake, and body weight were recorded in patient diaries. *C*_min_ samples were defined as those taken between 8 and 16 h after the prior dose.

#### Response to treatment

The response was considered optimal when a MMR was achieved within 12 months of nilotinib treatment [1, 42]. MMR was defined as a BCR-ABL transcript level of ≤ 0.1% on the International Scale. For patients already on treatment at entry, the time until MMR was obtained retrospectively from their medical files.

#### Questionnaires

At study entry, patients completed a self-administered composite questionnaire including demographic characteristics (date of birth, gender, education, living status, and working status), use of St. John’s wort, use of grapefruit (juice), quality of life (Short Form-12 Health Survey [SF-12]), side effects, and beliefs about medication in general (Beliefs about Medicines Questionnaire [BMQ]-General) and specifically about nilotinib (BMQ-Specific) [49, 50]. The questionnaire at 3, 6, and 12 months also included the MARS-5 and questions on nilotinib intake in relation to food intake, use of tools to prevent forgetting, illness perception (Brief Illness Perception Questionnaire [Brief IPQ]), and information satisfaction (Satisfaction with Information about Medicines Scale [SIMS])[51, 52]. Education was assessed as the highest level completed, and dichotomized into higher education (higher general secondary education or above) and lower education. Living status was categorized as living alone or not living alone, and work status as having paid work or not. Questions on side effects were based on literature data and concerned specific nilotinib toxicities occurring in > 10% of patients (headache, nausea, rash, itching, myalgia, fatigue) [53]. Answers were scored on a 5-point Likert scale (0 = not at all to 4 = very much). Symptoms scored as “3 = a lot” or “4 = very much” were considered “severe.”

#### Disease and treatment characteristics

Information on disease characteristics, medical history, co-medication, and nilotinib treatment was obtained from the patient’s medical file and pharmacy dispensing record.

### Statistical analysis

Differences in patient and clinical characteristics between the four groups were tested (continuous variables by Mann-Whitney tests and categorical variables by chi-square tests). Adherence was summarized by median and mean PDC (MEMS), AR (pill count), and MARS-5 scores, and the number of nonadherent patients according to various adherence cut-off points. Agreement between the adherence measures was examined using intra-class correlation coefficient (ICC, two-way mixed effects model) comparing MEMS (% PDC) with pill count (% AR) and Cohen’s Kappa comparing MEMS and pill count (dichotomized at thresholds of 90% and 95%) with MARS-5. Change over time in dichotomized MARS-5 scores was assessed using generalized estimating equation (GEE) and univariable logistic regression was used to identify variables (one by one) related to self-reported nonadherence (MARS-5 < 25 at 12 months). Variables related to incorrect intake of nilotinib under fasting conditions were identified using logistic regression (baseline characteristics) and GEE (repeatedly measured variables).

Individual measures of exposure were obtained using averaged *C*_min_ values for individual patients based on the measurements obtained at baseline, 3, 6, and 12 months. Intra-patient variability in nilotinib *C*_min_ was analyzed in patients who had ≥ 2 analyzed samples available and expressed as the average coefficient of variation (CV%). Missing *C*_min_ values were imputed with the mean of the observed *C*_min_ values for the corresponding dose. Univariable linear regression was used to identify variables (one by one) related to nilotinib *C*_min_. GEE was used to identify whether the occurrence of side effects was related to observed nilotinib *C*_min_ values (without imputation) obtained at baseline, 3, 6, and 12 months.

Comparison of adherence (continuous) and *C*_min_ values (either grouped into quartiles [Q1 vs. Q2–Q4] or based on a previously reported [13] threshold concentration of 490 μg/L) among response groups (time to MMR after start of nilotinib treatment ≤ 12 months vs. > 12 months, stratified by line of treatment) was performed using Mann-Whitney tests and Fisher’s exact tests. Time to MMR was assessed using the Kaplan Meier method with logrank test. In these outcome analyses, the mean of each measure of adherence and *C*_min_ values was used. For all analyses, a two-tailed significance level of 0.05 was used. *p* values below this level were considered statistically significant. All statistical analyses were performed using SPSS version 22 for Windows (IBM Corp, Armonk, NY, USA).

## Results

### Study sample

**Fig. 1:**
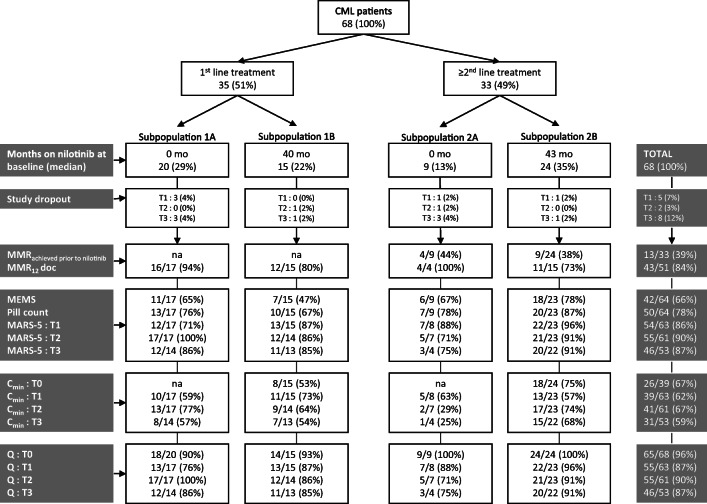
RAND study flow chart

A total of 68 patients (mean age 57.5 ± 15.0 years, 49% female) were included. Thirty-five patients (51%) received first-line nilotinib treatment, of whom 20 (29%) started treatment (subpopulation 1A) and 15 (22%) already were on treatment at baseline with a median duration of 40 months (range 7–51) (subpopulation 1B). Thirty-three patients (49%) received second/third-line nilotinib treatment, of whom 9 (13%) started treatment (subpopulation 2A) and 24 (35%) already were on treatment at baseline with a median duration of 43 months (range 7–51) (subpopulation 2B). Fifteen patients (22%) discontinued nilotinib treatment during the study period due to side effects (*n* = 6), progression (*n* = 4), entering treatment-free remission (*n* = 4), or death (*n* = 1). Their characteristics (demographics, medical history, nilotinib dose) did not differ from those of patients who completed follow-up. Five patients dropped out before assessment at 3 months, two patients before 6 months, and eight patients before 12 months. Figure 1 provides information on the number of patients for whom data are available.

### Patient characteristics

Patients’ characteristics are presented in Table 1. There were no statistically significant differences (*p* > 0.05) with regard to patient demographics, medical history, and nilotinib dose between the four subpopulations, except for a small difference in the median number of co-medications between subpopulations 1A/1B and 2A/2B (1 vs. 3, *p* = 0.043). Most patients (49/68, 72%) used the recommended dose of 300 mg BID. The other patients used a dose of 150 mg BID (*n* = 5), 400 mg BID (*n* = 9), 300 mg QD (*n* = 2), and 400 mg QD (*n* = 3). In three patients, the dose was reduced during the study period. The percentage of patients with co-medication was 73%. No patient reported using St. John’s wort. Based on the declared co-medications, there were no drug-drug interactions.

**Table 1:** Patient demographics and clinical status at baseline

		1st line treatment *n* = 35	≥2nd line treatment *n* = 33
Patient demographics			
Age, mean ± SD (years)		55.7 ± 15.0	59.3 ± 14.9
Female gender, *n* (%)		15 (43%)	18 (55%)
Higher level of education, *n* (%)		7 (20%)	12 (36%)
Living alone, *n* (%)		7 (20%)	2 (6%)
Employed, *n* (%)		18 (51%)	12 (36%)
Medical history			
History of other malignancy, *n* (%)		7 (20%)	7 (21%)
Presence of comorbidity, *n* (%)		16 (46%)	22 (67%)
Co-medication, range, median (IQR) *		0–12, 1 (0–4)	0–11, 3 (1–5)
No. of patients with ≥ 1 co-medication		22 (63%)	28 (85%)
History of CML and treatment			
Years since CML diagnosis, range, median (IQR)		1–4, 3.4 (1.2–3.9)^a^	1–22, 7.6 (5.0–10.5)
Prior TKI treatment and outcome, n (%)			
Imatinib		NA	32 (97%)
Failure			22 (69%)
Intolerance			9 (28%)
Unknown			1 (3%)
Dasatinib		NA	12 (36%)
Failure			4 (33%)
Intolerance			8 (66%)
Disease parameters at baseline			
Hematological response, *n* (%)			
Too early to judge		20 (57%)	-
CHR		13 (37%)	29 (88%)
No CHR		2 (6%)	3 (9%)
Not documented		-	1 (3%)
Molecular response, *n* (%)			
Too early to judge		20 (57%)	-
MR^4^ (BCR-ABL ≤ 0.01%)		10 (29%)	10 (30%)
Major MR (BCR-ABL ≤ 0.1%)		1 (3%)	9 (27%)
BCR-ABL > 0.1%		3 (9%)	10 (30%)
Not documented		1 (3%)	4 (12%)
Nilotinib treatment at baseline			
Months on nilotinib, range, median (IQR)		7–51, 40 (15–46)^a^	4–92, 43 (25–55)^a^
Dose	300 mg/day, *n* (%)	2 (6%)	5 (15%)
	400 mg/day, *n* (%)	-	3 (9%)
	600 mg/day, *n* (%)	32 (91%)	17 (52%)
	800 mg/day, *n* (%)	1 (3%)	8 (24%)

*Abbreviations*: *CHR*, complete hematological response; *CML*, chronic myeloid leukemia; *IQR*, interquartile range; *MR*, molecular response; *NA*, not applicable; *SD*, standard deviation; *TKI*, tyrosine kinase inhibitor

*Difference between groups significant (*p* ≤ 0.05)

^a^Patients already being on treatment at baseline, *n* = 15 (1st line) *n* = 24 (**≥** 2nd line)

Patients’ SF-12 scores for physical health measured at 6 months ranged from 17.1 to 58.4 (standardized scale 0–100 [Supplementary Table S1]). The median and mean scores were 45.4 and 44.1 ± 11.5, respectively. The SF-12 scores for mental health ranged from 27.3 to 61.8, with median and mean scores of 40.0 and 41.8 ± 6.9, respectively. About 75% of the patients experienced fatigue, which was severe in 20% (Supplementary Table S1). Rash, itching, and myalgia were reported by 43%, 59%, and 54% of the patients, respectively, and 8%, 8%, and 6% indicated these symptoms as severe. Headache and nausea were less common and generally mild.

The BMQ scores indicated that patients had strong beliefs about the necessity of taking nilotinib (20.5 ± 3.4) and low concerns about CML and the potential adverse effects of nilotinib (12.9 ± 3.4). The majority of patients had an accepting (65%) or ambivalent (30%) attitude towards nilotinib (Supplementary Table S1). Regarding illness perception, the high medians on the timeline (10/10), treatment control (10/10), and coherence (8/10) subscales indicate that patients were fully aware of the lifelong duration of CML, strongly believed in the effectiveness of nilotinib, and felt a strong coherent understanding of CML. The low median on the emotional response (3/10) subscale indicated that patients perceived only little emotional distress from CML.

### Adherence to nilotinib treatment

Data on adherence to nilotinib treatment are shown in Table 2. Adherence by means of MEMS was available for 42 patients, and monitored over a mean of 307 ± 113 days. Eight patients were not willing to use MEMS (as it would disturb their daily routines too much), three patients did not return their MEMS, and eleven patients returned MEMS with no data (either due to MEMS malfunction or patient refusal). Four patients discontinued nilotinib treatment within 4 days from study entry. The median and mean PDC from baseline to follow-up were 99.0% and 95.7 ± 8.5%, respectively (from 54.6 to 100%). Five patients (5/42, 12%) covered less than 90% of days observed. Adherence by means of pill count could be determined for 50 patients. For the remaining patients, the pill count was missing (*n* = 9), pharmacy data were incomplete (*n* = 5), or treatment was stopped within 4 days from study entry (*n* = 4). The median and mean AR were 99.8% and 98.3 ± 8.7%, respectively (from 67.3 to 125.6%). Five patients (5/50, 10%) had an AR < 90%. Five patients (5/50, 10%) had an AR > 105%.

**Table 2:** Adherence to nilotinib treatment

		Mean ± SD		Median (IQR)		Range	
Adherence by means of MEMS (*n* = 42)							
PDC, from baseline to follow-up		95.7 ± 8.5		99.0 (95.6–99.6)		54.6–100	
100%		6 (14.4%)					
≥ 95–< 100%		27 (64.3%)					
≥ 90–< 95%		4 (9.5%)					
< 90%		5 (11.9%)					
Adherence by means of pill count (*n* = 50)							
AR, from baseline to follow-up		98.3 ± 8.7		99.8 (96.4–101.6)		67.3–125.6	
> 105%		5 (10.0%)					
≥ 95%–≤ 105%		35 (70.0%)					
≥ 90–< 95%		5 (10.0%)					
< 90%		5 (10.0%)					
Adherence by means of MARS-5 (*n* = 56)							
MARS-5 Sum Score (5–25)		24.5 ± 1.0		25 (24–25)		21–25	
MARS-5 Sum Score < 25		18 (32.1%)					
Agreement of adherence assessment methods^a^							
		Pill count (continuous) (*n* = 36)					
		ICC	*p*				
MEMS (continuous)		0.14	.801				
		MARS-5 (dichotomous, < 25 vs. 25) (*n* = 34^mems^; *n* = 38^pill count^)					
		*K*	*p*				
MEMS (dichotomous)	< 90% vs. ≥ 90%	0.04	.778				
	< 95% vs. ≥ 95%	0.41	.014				
Pill count (dichotomous)	< 90% vs. ≥ 90%	− 0.03	.854				
	< 95% vs. ≥ 95%	− 0.04	.782				

*Abbreviations*: *AR*, adherence rate; *MARS-5*, Medication Adherence Report Scale, at ≥ 12 months of treatment; *MEMS*, Medication Event Monitoring System; *PDC*, proportion of days covered; *SD*, standard deviation; *IQR*, interquartile range; *ICC*, intra-class correlation coefficient; *K*, Kappa

^a^Analyses of continuous outcomes using ICC and dichotomous outcomes using Cohen’s Kappa

The percentage of patients reporting nonadherence on any of the five MARS-5 statements after 12 months was 32%. Median and mean MARS-5 scores of the nonadherent patients were 24 and 23.3 ± 1.0, respectively. The most common statement was “I forget to take it” varying from 17 to 26%. The statements “I alter the dose,” “I stop taking it for a while,” and “I decide to miss out on a dose” were reported by ≤ 6%. Seventeen patients (27%) did not always correctly take nilotinib under fasting conditions (i.e., < 60 min before or < 120 min after food intake). No patient reported to have taken nilotinib simultaneously with food or to have used grapefruit (juice) during nilotinib treatment. Most patients (87%) reported to have used a tool to remind them of the scheduled nilotinib intake (e.g., fixed place/moment, alarm, diary, support from family/friends).

Agreement between MEMS (% PDC) and pill count (% AR) was poor, with an ICC of 0.14 (*p* = 0.801) (Table 2). Regarding dichotomized MARS-5 scores (25 vs. < 25), only adherence by means of MEMS dichotomized at a threshold of 95% showed moderate agreement, with Cohen’s Kappa varying from 0.29 to 0.47 (*p* < 0.1).

The number of patients reporting nonadherence (MARS-5 data) increased over time for patients starting nilotinib first-line treatment (subpopulation 1A) from 8% after 3 months of treatment to 33% after 12 months. In the other three subpopulations, the percentage of patients ranged from 27 to 37%. This group-by-time interaction effect was close to significance (*p* = 0.066). Self-reported nonadherence was not associated with differences in disease and treatment characteristics, occurrence of side effects, and SF-12, Brief IPQ, BMQ-Specific, and SIMS scores (Supplementary Table S2). Regarding demographics, only gender was associated with self-reported nonadherence. Female patients more often reported nonadherence (44% vs. 13% in male patients, OR 5.1, 95%CI 1.2–22.2, *p* = 0.029). As only few patients had an PDC (MEMS data) or AR (pill count data) below 90%, no factors could be analyzed for their relationship with nonadherence as assessed by MEMS or pill count. No association was found between all variables and incorrect intake of nilotinib under fasting conditions (Supplementary Table S2).

### Nilotinib *C*_min_

A total of 178 DBS samples were collected by 61 patients. Thirty-five samples (19.7%) were rejected because the spot size was too small for analysis. Six samples (3.4%) had been obtained within 8 h of nilotinib intake and therefore were excluded as *C*_min_ [48]. As the result, 137 *C*_min_ values from 56 patients were obtained (Table 3). Forty-two patients (72%) had ≥ 2 analyzed samples available, and the %CV in *C*_min_ amounted to 25 ± 19% (median 20%; range 1–91%). No association was found between all measures of adherence and nilotinib *C*_min_ (Table 3). Age, gender, body weight, incorrect intake of nilotinib under fasting conditions, and intra-patient variability were also not significantly related to the nilotinib *C*_min_. A positive linear association between nilotinib dose and *C*_min_ was found (*β* = 0.44, *p* < 0.001).

**Table 3:** Associations with nilotinib *C*_min_

**Observed nilotinib** ***C***_**min (**_**μg/L)**		**Mean ± SD**	**Median (IQR)**	**Range**
400 mg BID (*n* = 25)		1497 ± 597	1253 (1054–2053)	342–2540
300 mg BID (*n* = 85)		1021 ± 472	954 (654–1274)	196–2413
150 mg BID (*n* = 15)		835 ± 412	656 (548–1042)	390–1793
400 mg QD (*n* = 6)		536 ± 57	552 (517–570)	422–574
300 mg QD (*n* = 6)		567 ± 110	589 (450–658)	418–702
**Associations with nilotinib** ***C***_**min**_^**a**^		***β***	***p***	
Age		0.08	.534	
Female gender		0.05	.687	
Body weight		0.03	.982	
Dose		0.44	*< .001*	
CV%		0.23	.154	
Incorrect intake under fasting conditions^b^	T1	− 0.07	.712	
	T2	− 0.01	.949	
	T3	0.21	.297	
Adherence to nilotinib^b^	MEMS	− 0.05	.763	
	Pill count	− 0.13	.396	
	MARS-5 T1	0.13	.466	
	MARS-5 T2	0.05	.753	
	MARS-5 T3	− 0.14	.472	

*Abbreviations*: *BID*, twice daily; *QD*, once daily; *C*_*min*_, trough plasma concentration; *CV%*, coefficient of variation; *MARS-5*, Medication Adherence Report Scale; *MEMS*, Medication Event Monitoring System; *SD*, standard deviation; *IQR*, interquartile range. Significant relations are shown in *italic* (*p* < 0.05)

^a^BID dosing, *C*_min_ samples were taken between 8 and 16 h after the prior dose (*n* = 125)

^b^Incorrect intake of nilotinib under fasting conditions and MARS-5 was related to observed *C*_min_ values at 3 (T1), 6 (T2), and 12 (T3) months from baseline

The relationship between the nilotinib *C*_min_ value and patient-reported side effects is presented in Table 4. The mean *C*_min_ was significantly higher in patients who reported severe itching (*p* = 0.012) and fatigue (*p* = 0.023). With regard to fatigue, there was a significant difference in *C*_min_ between patients reporting any severity fatigue and those without fatigue (*p* = 0.007). The median nilotinib dose was 600 mg in all side effect groups. No relationship with other side effects was found.

**Table 4:** Nilotinib *C*_min_ and patient-reported side effects of nilotinib^a^

	None		Mild^b^		Severe^b^		*p*	
	*n*	*C*_min_ (μg/L) median (IQR)	*n*	*C*_min_ (μg/L) median (IQR)	*n*	*C*_min_ (μg/L) median (IQR)	Any	Severe
Headache	91	970 (618–1288)	40	905 (625–1254)	1	2413	.964	-
Nausea	104	945 (615–1249)	27	1210 (750–1798)	1	648	.151	-
Rash	75	964 (598–1338)	45	966 (719–11620	12	1157 (764–1540)	.418	.746
Itching	61	954 (565–1283)	61	923 (740–1243)	11	1260 (1021–1800)	.938	*.012*
Myalgia	56	857 (574–1219)	62	1031 (765–1380)	12	840 (658–1553)	.387	.399
Fatigue	32	777 (561–1087)	72	974 (644–1373)	25	1160 (787–1468)	*.007*	*.023*

*Abbreviations*: *C*_*min*_, trough plasma concentration; *IQR*, interquartile range. Significant relations are shown in *italic* (*p* < 0.05)

^a^Analyses using generalized estimated equations (GEE)

^b^Side effects scored as “a little bit”/”rather” were considered “mild”; side effects scored as “a lot”/”very much” were considered “severe”

### Molecular response to nilotinib treatment

For patients on first-line nilotinib treatment (subpopulations 1A/1B), the 1-year MMR rate was 71% (20/28) (note, four missing). The median and mean times to MMR_12_ were 6.1 and 5.9 ± 3.2 months, respectively (range 1.2–12.9 months). Two patients discontinued nilotinib treatment before achieving a MMR due to side effects and one patient died. For patients on second/third-line nilotinib treatment (subpopulations 2A/2B), the 1-year MMR rate was 47% (7/15) (note, four missing). The median and mean times to MMR_12_ were 3.7 and 5.8 ± 4.4 months, respectively (range 2.6–12.4 months). One patient discontinued nilotinib treatment before achieving a MMR due to side effects. Thirteen patients had achieved a MMR prior to nilotinib treatment, of whom seven patients a deep MR^4^.

Patients who achieved a MMR within 12 months after having started nilotinib treatment had similar adherence rates (mean PDC 95.0 ± 8.7%) as those who failed to obtain a MMR within 12 months of treatment (mean PDC 94.6 ± 11.9%, *p* = 0.683) (Table 5). Among patients treated with either first-line or second/third-line nilotinib, no significant differences in mean adherence were found between the response groups. There were no significant differences between response groups in self-reported adherence (MARS-5) (data not shown).

**Table 5:** Adherence, *C*_min_, and molecular response to nilotinib treatment

**Adherence and molecular response to nilotinib treatment**						
	Adherence using MEMS			Adherence using pill count		
	*n*	Mean ± SD	*p*	*n*	Mean ± SD	*p*
All patients			.683			.927
Optimal response	15	95.0 ± 8.7%		21	96.9 ± 11.0%	
Suboptimal response	11	94.6 ± 11.9%		12	99.0 ± 2.6%	
Subpopulation 1A/1B (1st line treatment)			.441			.444
Optimal response	11	97.0 ± 5.0%		14	97.3 ± 9.9%	
Suboptimal response	5	93.9 ± 13.1%		6	100.7 ± 1.2%	
Subpopulation 2A/2B (≥ 2nd line treatment)			.352			.731
Optimal response	4	88.0 ± 22.3%		7	96.0 ± 13.8%	
Suboptimal response	6	95.9 ± 3.7%		6	97.3 ± 2.6%	
**Nilotinib** ***C***_**min**_ **and molecular response to nilotinib treatment**						
	1-year MMR rate according to nilotinib *c*_min_					*p*
	Q1	Q2	Q3		Q4	Q1 vs. Q2–Q4
All patients	56% (5/9)	88% (7/8)	75% (12/16)		30% (3/10)	.706
Subpopulation 1A/1B (1st line treatment)	71% (5/7)	100% (5/5)	73% (8/11)		40% (2/5)	.999
Subpopulation 2A/2B (≥ 2nd line treatment)	0% (0/2)	75% (2/3)	80% (4/5)		20% (1/5)	.467

*Abbreviations*: *C*_*min*_, trough plasma concentration; *MEMS*, Medication Event Monitoring System; *SD*, standard deviation; *Q*, quartile

Optimal and suboptimal responses were defined as time to major molecular response ≤ 12 months and > 12 months, respectively. *C*_min_ quartiles were Q1 (< 635 μg/L), Q2–Q3 (635–< 1346 μg/L), Q4 (≥ 1346 μg/L)

Among the evaluable patients, the MMR rate was not significantly different between *C*_min_ quartiles (Table 5). The median time to MMR_12_ was 6.4 (Q1, *n* = 5), 6.7 (Q2, *n* = 7), 4.3 (Q3, *n* = 12), and 2.8 months (Q4, *n* = 3), and was not statistically different among the quartile groups (*p* = 0.098). Nine patients (16%) had a *C*_min_ below the threshold concentration of 490 μg/L at any time (range 196–467), of whom six patients (11%) once and two patients (4%) twice. One patient (2%) had four *C*_min_ values below 490 μg/L. These patients did not differ in time to MMR_12_ from those with *C*_min_ above 490 μg/L.

## Discussion

This comprehensive observational study in CP-CML patients using nilotinib aimed to obtain insight into their adherence to treatment and its influence on drug exposure and treatment outcomes. The median adherence to nilotinib was high (≥ 99%) and adherence lower than 90% was rare. Nevertheless, after 12 months of treatment, about a third of the patients reported occasional nonadherence. In line with the high belief of patients in the necessity of taking nilotinib, forgetting to take a dose was more prevalent than intentionally adjusting or skipping doses. Although the intra-patient variability was high, nilotinib C_min_ values were generally above the therapeutic target in 95% of patients. Patients reported a variety of side effects, of which fatigue was most frequent. The mean *C*_min_ was higher in patients who reported severe itching and fatigue.

Several studies assessing patients’ adherence to nilotinib using objective measures reported generally lower rates of adherence [23–35]. However, various methodological issues (i.e., study design and setting, follow-up period, assessment measure) limit comparison across studies [37, 54, 55]. Most of the studies were retrospective using claims data or pharmacy refill data [23–32, 35]. The sample sizes of these studies were larger and its data gathered unobtrusively without patient involvement. In the present study, nonadherent patients may have been less willing to participate which may lead to an over-estimation of adherence. In addition, studies using claims data or pharmacy refill data may underestimate the level of adherence because dose reductions are generally not taking into account in time. As yet prospectively obtained data are limited to those of ten patients participating in the TAKE-IT study investigating adherence by means of MEMS in CP-CML patients using imatinib, dasatinib, or nilotinib [36, 56]. Although the median overall adherence (97.5%) for all TKI was similar to that found in the present study, adherence was somewhat lower in patients treated with nilotinib as compared with imatinib and dasatinib.

In accordance with the findings of a recent review comparing MEMS with pill count and self-report [54], in the present study, there was poor agreement between adherence as assessed by these measures. In 81% of the studies reviewed, a significant difference between the rates obtained by applying each of these measures was found [54]. Clearly, each measure obtains its data differently and identifies different components of adherence. Since MEMS provides detailed, objective data on adherence, it is most often considered to be the gold standard [57]. However, the use of this system is known to influence adherence [57]. Moreover, as the MEMS solely records the opening of the container, evidence that (the full dose of) medication is actually ingested is not provided [57]. The pill count method is less interfering than the use of MEMS but fails to provide insight into adherence patterns [55, 58]. Likewise, it does not provide evidence that medication is actually taken. Subjective measures generally provide explanations for nonadherence; however, results can be biased by patients giving false information [55, 58]. Therefore, a combination of objective and subjective measures is considered the best solution to assess medication adherence [55, 58].

Although the adherence over the full study period was high, occasional nonadherence measured with MARS-5 increased in the first year of nilotinib treatment to a third of the patients. This finding is consistent with the results of other studies [39, 59, 17]. Over time, patients become accustomed to living with a chronic disease and taking medication daily. Due to apparently successful treatment, patients increasingly participate in social and occupational activities. However, this often interferes with their daily routines which makes patients more easily to forget or skip a dose [39, 59, 60, 17]. On the other hand, intentional nonadherence is mostly due to perceptual barriers (i.e., beliefs) [61, 62]. In the present study, forgetting to take nilotinib was found to be more prevalent than intentionally skipping or adjusting a dose and could be explained by the strong beliefs of patients in the necessity of taking nilotinib. The seriousness of the disease and clinical importance of TKI treatment are likely to reflect a patients' perceived need for treatment [63]. HCP should encourage CP-CML patients using nilotinib to use practical aids (such as alarm devices and pill boxes) in order to avoid forgetting their medication as part of measures that prevent adherence to decline over time. In line with the results of a recently published worldwide patient survey by the CML Advocates Network [39], in the present, study female patients reported nonadherence more often than male patients.

A quarter of the patients did not consistently follow the recommendation to take nilotinib at least 1 h before or 2 h after a meal [53]. Food substantially increases nilotinib bioavailability [16, 64] which may cause adverse events [12]. Clearly, patients should be supported in correctly taking nilotinib in order to avoid potentially hazardous nilotinib concentrations. On the other hand, by applying a lower dose, the food-dependent bioavailability of nilotinib can be used to improve intake conditions [64]. As this simplified dosing regimen is likely to promote adherence and quality of life, its usefulness in daily practice should be further explored [64].

The prevalence rates of headache, nausea, rash, itching, and myalgia found in the present real-world study were essentially similar to those found in the ENESTnd trial [65]. Fatigue was reported by about one-fifth of the patients in this trial [65]. In contrast, in the present study, this debilitating side effect was reported by about three quarters of the patients. The lower prevalence of fatigue in the ENESTnd trial [65] might be explained by the propensity of HCP to underestimate symptomatic, subjective side effects [66, 67]. Our findings are in line with those of other studies providing data on patient-reported side effects [68–70]. Although nilotinib side effects are generally mild, they do adversely impact the patients’ quality of life [71, 68, 72]. Considering the long duration of CML treatment and increased risk of treatment interruption, discontinuation, or switching due to side effects, it is important to actively inquire after (perceived) side effects and subsequently support patients in mitigating their effects [73]. Remarkably, the mean *C*_min_ of nilotinib was higher in patients reporting severe itching and fatigue. As yet higher nilotinib *C*_min_ have only been associated with the occurrence of all-grade elevations in total bilirubin and lipase levels and increases in QTc changes [12, 13]. Therapeutic drug monitoring (TDM) may prevent the emergence of potentially hazardous nilotinib plasma concentrations. On the other hand, in certain patients with higher *C*_min_, the dose might be reduced on the basis of TDM without compromising treatment efficacy.

CML patients vary greatly in their responses to treatment. In the present study, 71% of the patients on first-line nilotinib treatment achieved a 1-year MMR rate. This is higher than the 55% rate reported in the 5-year update of the ENESTnd trial [65]. In this study of highly adherent patients, we could not identify whether a lack of adherence contributed to a lack of response. The same applied for nilotinib blood levels, which were generally above the minimum therapeutic target. Apparently, in this group of patients, the incapacity to achieve a MMR during 1 year of treatment seemed not related to nonadherence or inadequate nilotinib blood levels. However, the underpowering of the study due to the unavoidable heterogeneity of its study population precludes any definitive conclusions.

The present study has some strengths and limitations. A major strength is its prospective design that provides a unique and complete survey of nilotinib treatment in daily practice. A wide range of variables was longitudinally collected by means of questionnaires, blood sampling, adherence measures, and data retrieved from medical files and pharmacy records. To our knowledge, this is the first study in which these real-life nilotinib data have been evaluated together. Another strength is the assessment of medication adherence, which was prospectively implemented using both objective and subjective measures. Also, it includes a long follow-up of 12 months. Unfortunately, in the Netherlands, the number of patients treated with nilotinib is relatively small resulting in a poor initial accrual of newly diagnosed CP-CML patients. Consequently, the study protocol had to be amended to include both patients already treated with nilotinib treatment and those with prior TKI treatment. Selection bias may have occurred, as nonadherent patients may have been less willing to participate. In addition, patients willing to participate may be more attentive to treatment, resulting in optimal responses, adherence, and nilotinib blood levels in our group of patients. Caution should be used when generalizing the findings to other CML patients. The inability to collect adherence data at treatment onset in patients already on nilotinib treatment (subpopulations 1B/2B) may have biased the results of the adherence-outcome analyses as mean adherence values were used, whereas our analyses showed that self-reported nonadherence increased over time in subpopulation 1A. Another limitation is the amount of missing data. In order to minimize their influence on outcomes, we have decided to limit imputation to the exposure-outcome and exposure-factors analyses.

## Conclusion

Although in most patients in the present study the extent of nonadherence to nilotinib appeared not to be clinically relevant with respect to achieving an optimal response, it is clear that a considerable number of patients experienced difficulties in adhering to the recommended twice daily fasted dosing regimen. Current clinical practice may be improved by fostering the intention to adhere and by encouraging patients to use practical aids that are particularly relevant to avoid the occurrence of unintentional nonadherence. Since adherence decreases by treatment duration, interventions aimed at long-term correct use of nilotinib are also relevant. Furthermore, HCP should inquire after (perceived) side effects and take adequate measures to mitigate these effects, in particular the occurrence of fatigue. TDM whether or not in combination with dose reduction may be considered in order to avoid unnecessary high blood levels causing severe side effects.

## Electronic supplementary material

ESM 1 (DOCX 23 kb)
