# Supplementary material for: Response and Adherence to Nilotinib in Daily practice (RAND study): an in-depth observational study of chronic myeloid leukemia patients treated with nilotinib
Source: Eur J Clin Pharmacol. 2020 Jun 2;76(9):1213–26. doi: 10.1007/s00228-020-02910-3 (PMC7419465; doi:10.1007/s00228-020-02910-3)
Supplement: Supplementary file 1 — (DOCX 23 kb) [file 228_2020_2910_MOESM1_ESM.docx]

| **Supplementary Table S1** Quality of life, attitude towards disease and nilotinib | | |
| --- | --- | --- |
|  | mean ± SD | median (IQR) |
| **Quality of life (SF-12)** |  |  |
| Physical health (0-100) | 44.1 ± 11.5 | 45.4 (35.9 – 55.0) |
| Mental health (0-100) | 41.8 ± 6.9 | 40.0 (36.8 – 46.7) |
| **Side effects^a^** |  |  |
| Headache, % mild, % severe | 35.8%, 1.9% |  |
| Nausea, % mild, % severe | 20.4%, 1.9% |  |
| Rash, % mild, % severe | 35.2%, 7.4% |  |
| Itching, % mild, % severe | 51.9%, 7.4% |  |
| Myalgia, % mild, % severe | 48.1%, 5.8% |  |
| Fatigue, % mild, % severe | 54.9%, 19.6% |  |
| **Beliefs about medicines (BMQ)** |  |  |
| Necessity beliefs (5-25) | 20.5 ± 3.4 | 20.5 (18.8 – 23.3) |
| Concern beliefs (5-25) | 12.9 ± 3.4 | 12.5 (10.0 – 15.3) |
| Necessity-Concerns differential (-20 till +20) | 7.6 ± 4.8 | 8.0 (4.8 – 11.0) |
| Harm beliefs (4-20) | 9.1 ± 2.4 | 9.0 (8.0 – 11.0) |
| Overuse beliefs (4-20) | 9.9 ± 2.5 | 10.0 (8.8 – 12.0) |
| Attitudinal profiles, % |  |  |
| Ambivalent | 64.8% |  |
| Accepting | 29.6% |  |
| Indifferent | 5.6% |  |
| Skeptical | 0.0% |  |
| **Illness perception (Brief IPQ)** |  |  |
| Consequences (0-10) | 4.8 ± 2.8 | 5.0 (2.0 – 7.0) |
| Time line (0-10) | 9.0 ± 1.7 | 10.0 (8.3 – 10.0) |
| Personal control (0-10) | 4.9 ± 3.4 | 5.0 (1.0 – 8.0) |
| Treatment control (0-10) | 9.2 ± 1.1 | 10.0 (8.0 – 10.0) |
| Identity (0-10) | 4.3 ± 3.0 | 5.0 (1.0 – 7.0) |
| Concern (0-10) | 4.8 ± 2.8 | 5.0 (2.0 – 7.0) |
| Coherence (0-10) | 8.0 ± 2.0 | 8.0 (7.0 – 9.3) |
| Emotional response (0-10) | 3.5 ± 2.8 | 3.0 (1.0 – 6.0) |
| **Information satisfaction (SIMS)** |  |  |
| Overall satisfaction (0-17) | 13.9 ± 3.4 | 15.0 (12.0 – 17.0) |
| Subscale Action and usage (0-9) | 8.1 ± 1.4 | 9.0 (7.0 – 9.0) |
| Subscale Potential problems (0-8) | 5.9 ± 2.4 | 7.0 (4.0 – 8.0) |
| Abbreviations: SF-12, Short-Form Health Survey; BMQ, Beliefs about Medicines Questionnaire; Brief IPQ, Brief Illness Perception Questionnaire; SIMS, Satisfaction with information about Medicines Scale; SD, standard deviation; IQR, interquartile range.  a Side effects scored as ‘a little bit’/‘rather’ were considered ‘mild’, side effects scored as 'a lot'/'very much' were considered 'severe'. | | |

| **Supplementary Table S2** Associations with self-reported nonadherence (MARS-5 <25) and incorrect intake of nilotinib under fasting conditions^a^ | | | | | |
| --- | --- | --- | --- | --- | --- |
|  | | **MARS-5 <25** | | **Incorrect intake** | |
|  | | OR (95%BI) | *P* | OR (95%BI) | *P* |
| Age <55 years | | 1.5 (0.4-5.6) | .540 | 1.0 (0.3-3.4) | .947 |
| Female gender | | 5.1 (1.2-22.2) | **.029** | 0.4 (0.1-1.4) | .160 |
| Low education | | 1.7 (0.4-10.0) | .544 | 5.0 (0.8-10.0) | .074 |
| Living alone | | 0.2 (0.0-1.6) | .135 | 0.3 (0.1-1.5) | .147 |
| Employed | | 0.6 (0.2-2.2) | .428 | 1.0 (0.3-3.0) | .963 |
| History of other malignancy | | 0.3 (0.0-2.8) | .297 | 1.4 (0.4-5.5) | .610 |
| Presence of comorbidity | | 0.4 (0.1-1.6) | .193 | 1.1 (0.4-3.4) | .887 |
| ≥1 co-medication | | 1.1 (0.2-4.9) | .934 | 3.4 (0.7-16.9) | .136 |
| Years since CML diagnosis | | 1.0 (0.9-1.2) | .503 | 1.0 (0.9-1.1) | .892 |
| Duration of nilotinib treatment | | 1.0 (1.0-1.0) | .761 | 1.0 (1.0-1.0) | .694 |
| Second/third line treatment | | 0.8 (0.2-2.9) | .744 | 0.4 (0.1-1.4) | .160 |
| Quality of life^b^ | Physical health | 0.5 (0.1-2.8) | .440 | 1.3 (0.5-3.5) | .623 |
|  | Mental health | 1.0 (0.2-4.6) | .999 | 0.7 (0.3-1.5) | .328 |
| Patient-reported side effects | No. of side effects (0-6) | 1.1 (0.7-1.6) | .728 | 1.0 (0.9-1.0) | .361 |
| (any severity) | Headache | 2.5 (0.6-11.3) | .238 | 0.9 (0.3-2.6) | .865 |
|  | Nausea | 1.0 (0.3-4.1) | .975 | 1.3 (0.3-5.0) | .729 |
|  | Rash | 1.8 (0.5-6.6) | .376 | 2.2 (0.9-5.1) | .068 |
|  | Itching | 1.4 (0.4-5.1) | .609 | 1.3 (0.5-3.2) | .608 |
|  | Myalgia | 0.7 (0.2-2.4) | .539 | 1.6 (0.6-3.8) | .338 |
|  | Fatigue | 0.6 (0.2-2.3) | .460 | 1.5 (0.6-3.5) | .338 |
| Illness perception (Brief IPQ)^b^ | Consequences | 1.9 (0.5-7.6) | .360 | 0.6 (0.2-1.9) | .362 |
|  | Timeline | - | - | - | - |
|  | Personal control | 2.0 (0.5-8.7) | .356 | 1.6 (0.5-5.6) | .456 |
|  | Treatment control | 0.9 (0.2-4.3) | .934 | 0.9 (0.4-2.2) | .803 |
|  | Identity | 1.5 (0.4-6.0) | .541 | 1.3 (0.4-5.0) | .682 |
|  | Concerns | 1.6 (0.3-8.1) | .556 | 0.6 (0.3-1.5) | .297 |
|  | Coherence | 0.9 (0.2-3.9) | .849 | 0.4 (0.2-1.0) | .059 |
|  | Emotional response | 1.8 (0.5-7.1) | .404 | 1.1 (0.3-4.0) | .849 |
| Beliefs about nilotinib (BMQ-S) | Accepting | ref. |  | ref. |  |
|  | Ambivalent | 3.3 (0.8-13.9) | .103 | 0.9 (0.4-2.3) | .844 |
|  | Indifferent | - | - | 1.8 (0.2-17.4) | .627 |
|  | Skeptical | - | - | - | - |
| Information satisfaction (SIMS)^c^ | Overall satisfaction | 0.7 (0.2-2.6) | .538 | 0.7 (0.3-1.7) | .401 |
|  | Subscale Action and usage | 0.5 (0.1-2.5) | .375 | 0.7 (0.2-2.3) | .606 |
|  | Subscale Potential problems | 1.4 (0.4-5.3) | .592 | 0.6 (0.2-1.3) | .165 |
| Abbreviations: BMQ-S, Beliefs about Medicines Questionnaire Specific; Brief IPQ, Brief Illness Perception Questionnaire; MARS-5, Medication Adherence Report Scale; SIMS, Satisfaction with Information about Medicine Scale; SF-12, SF-12 Health Survey; OR, odds ratio; 95%CI, 95% Confidence Interval. Significant relations are shown in **bold** (p<0.05).  a Associations (one by one) between repeatedly measured variables (QoL, side effects, Brief IPQ, BMQ, SIMS) and incorrect intake of nilotinib were tested using generalized estimating equation analyses (GEE); the other factors were tested using logistic regression; b Dichotomized into the most adverse quartile versus the other three quartiles (reference), c Dichotomized into dissatisfaction <14/17, <8/9, <6/8 items (resp. overall satisfaction, action and usage, potential problems) versus satisfaction (reference). | | | | | |
